# Covalent Stabilization of the Iridium-Containing Oxyhydrides Sr_2_Mn_0.5_Ir_0.5_O_3.25_H_0.75_ and Sr_2_Mn_0.5_Ir_0.5_O_2.66_H_1.33_

**DOI:** 10.1021/acs.inorgchem.4c04057

**Published:** 2024-11-05

**Authors:** James I. Murrell, Michael A. Hayward

**Affiliations:** † Department of Chemistry, University of Oxford, Inorganic Chemistry Laboratory, South Parks Road, Oxford, OX1 3QR, United Kingdom

## Abstract

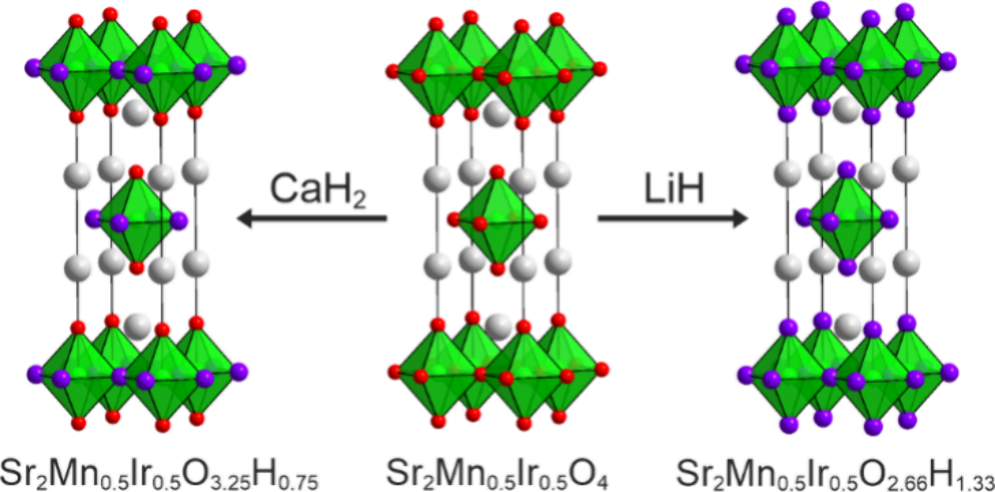

Reaction between Sr_2_Mn_0.5_Ir_0.5_O_4_ and CaH_2_ or LiH yields the iridium-containing oxyhydride phases Sr_2_Mn_0.5_Ir_0.5_O_3.25_H_0.75_ or Sr_2_Mn_0.5_Ir_0.5_O_2.66_H_1.33_, respectively. Analysis of Mn K-edge XANES data indicate the presence of Ir^3+^ centers in these oxyhydride phases, whose low-spin d^6^ configuration is consistent with the “covalent stabilization” of the metastable oxyhydride phases, as seen previously in analogous ruthenium and rhodium containing materials. Neutron powder diffraction data indicate the hydride ions are located exclusively within the “equatorial” anion sites of Sr_2_Mn_0.5_Ir_0.5_O_3.25_H_0.75_. In contrast, hydride ions are observed on both the equatorial and axial anion sites of Sr_2_Mn_0.5_Ir_0.5_O_2.66_H_1.33_. This highly unusual anion distribution is attributed to a combination of the strong *trans*-influence of Ir–H σ-bonds and the stabilization of *fac*-IrO_3_H_3_ centers by spin–orbit coupling effects. Magnetization data indicate that Sr_2_Mn_0.5_Ir_0.5_O_4_ and Sr_2_Mn_0.5_Ir_0.5_O_3.25_H_0.75_ adopt spin glass states at low temperature, behavior which is attributable to the cation disorder in Sr_2_Mn_0.5_Ir_0.5_O_4_ and the cation and anion disorder in Sr_2_Mn_0.5_Ir_0.5_O_3.25_H_0.75_. In contrast, magnetization data collected from Sr_2_Mn_0.5_Ir_0.5_O_2.66_H_1.33_ show no evidence of any magnetic phase transition down to 5 K, consistent with the dilution of the magnetic network by the introduction of diamagnetic Ir^3+^ on the formation of the oxyhydride phase.

## Abstract 2

Reaction between Sr_2_Mn_0.5_Ir_0.5_O_4_ and CaH_2_ or LiH yield the iridium-containing oxyhydride phases Sr_2_Mn_0.5_Ir_0.5_O_3.25_H_0.75_ or Sr_2_Mn_0.5_Ir_0.5_O_2.66_H_1.33_ respectively, which are stabilized by the presence of strong Ir−H σ-bonds.

## Introduction

Transition-metal oxyhydrides, mixed-anion phases which contain both O^2–^ oxide and H^–^ hydride anions, can exhibit bonding regimes and electronic structures which are qualitatively different to all-oxide analogues.^1^ The many contrasting features of oxide and hydride ions allow the behavior of oxyhydride phases to be rationally tuned by anion substitution. For example, the lower charge of H^1–^ hydride compared to O^2–^ oxide makes hydride-for-oxide anion substitution a reductive process; thus, transition-metal oxyhydrides tend to contain metal cations in usually low oxidation states (Co^1+^, V^3+^, Ru^2+^, Ni^1+^)^2−4^ compared to all-oxide analogues. In addition, the greater polarizability and lower electronegativity of hydride ions give M-H bonds a higher degree of covalency compared to the corresponding M-O bonds. This can also be seen in the related observation that hydride ions are stronger σ-donors and stronger field ligands than oxide ions, meaning that oxyhydride ligand sets can give rise to transition-metal d-orbital splitting arrangements, which are not easily achieved in all-oxide coordination environments. Furthermore, the lack of π-symmetry valence orbitals in hydride ions means they cannot bond to metal orbitals of π-symmetry (d_*xy*_, d_*xz*_, d_*yz*_ in octahedral coordination) which can dramatically affect the electronic dimensionality of anion-ordered oxyhydride phases such as SrVO_2_H.^3,5^ As a consequence, there is an enduring interest in the preparation of new transition-metal oxyhydride phases.

However, the preparation of transition-metal oxyhydrides is challenging. Principally this is because these phases are metastable at ambient pressure with respect to decomposition to form water, elemental transition metals and simple binary oxides (e.g., 2 LaSrCoO_3_H_0.7_ → La_2_O_3_ + 2 SrO + 0.7 H_2_O + 1.7 Co + 0.3 CoO). As a result, transition-metal oxyhydrides are only kinetically stable at ambient pressure.

Recently, we demonstrated that transition-metal oxyhydride phases can be kinetically stabilized via the formation of strong transition-metal-hydride σ-bonds. Initially this “covalent stabilization” strategy was observed in the cobalt phases LaSrCoO_3_H_0.7_ and Sr_3_Co_2_O_4.33_H_0.84_^2,6,7^ before being utilized to prepare Ru and Rh containing oxyhydrides such as LaSr_3_NiRuO_4_H_4_, LaSrCoRuO_3.2_H_1.9_ and La_0.5_Sr_1.5_Mn_0.5_Rh_0.5_O_3_H.^4,8,9^ Here we report the preparation of what we believe are the first iridium-containing oxyhydride phases, Sr_2_Mn_0.5_Ir_0.5_O_3.25_H_0.75_ and Sr_2_Mn_0.5_Ir_0.5_O_2.66_H_1.33_, in which strong Ir–H σ-bonds appear to stabilize the first 5d transition-metal-containing oxyhydride phase.

## Experimental Section

### Synthesis of Sr_2_Mn_0.5_Ir_0.5_O_4_

Samples of Sr_2_Mn_0.5_Ir_0.5_O_4_ were prepared by a high-temperature ceramic synthesis method. Suitable stoichiometric ratios of SrCO_3_ (99.99%), MnO_2_ (99.997%), and IrO_2_ (99.99%, dried for 2 h at 700 °C) were thoroughly ground together in an agate pestle and mortar, pressed into pellets and then heated in air at 1000 °C for 48 h within an alumina crucible. The samples were then reground, pressed into pellets, and reheated for 48 h periods between 1300 and 1385 °C until no further change was observed in powder X-ray diffraction data.

### Topochemical Reduction of Sr_2_Mn_0.5_Ir_0.5_O_4_

Samples of Sr_2_Mn_0.5_Ir_0.5_O_4_ were reduced by reaction with either LiH or CaH_2_. Initially, reactivity was assessed by grinding small samples of Sr_2_Mn_0.5_Ir_0.5_O_4_ (∼200 mg) with either 2 mol equivalents of CaH_2_ or 4 mol equivalents of LiH in an argon-filled glovebox. The resulting mixtures were then sealed in evacuated Pyrex ampules and heated as described below. Larger samples for characterization by neutron diffraction were prepared as described in the text below. ***CAUTION:** Reactions between binary metal hydrides (LiH, CaH_2_) and transition metal oxides generate H_2_ gas. It is essential to ensure sample containers are of sufficient volume to avoid hazardous pressure build ups.*

After the reaction, LiH-reduced samples were washed with methanol, under nitrogen, to remove the Li_2_O reaction by product and excess LiH, before being dried under vacuum. CaO was removed from CaH_2_-reduced samples by stirring the reaction products in glycerol for 12 h and then filtering the solid material before washing with dry methanol and drying under vacuum.

### Characterization

Reaction progress and initial structural characterization were performed by using laboratory X-ray powder diffraction (PXRD) data collected by using a Bruker D8 Advance diffractometer with Cu Kα radiation and a Lynx-eye PSD detector (Cu Kα radiation). Air-sensitive samples were measured in enclosed cells sealed under argon. High-resolution synchrotron powder X-ray diffraction (SXRD) data were collected using the I11 instrument at Diamond Light Source Ltd. Diffraction patterns were collected using Si-calibrated X-rays with an approximate wavelength of 0.825 Å from samples sealed in 0.3 mm diameter borosilicate glass capillaries. Neutron powder diffraction (NPD) data were collected using the GEM diffractometer at the ISIS neutron source from samples contained within vanadium cans sealed under an inert atmosphere. Rietveld refinement of powder diffraction data was performed using the TOPAS Academic software package (V6).^10^ Thermogravimetric analysis (TGA) measurements were performed by heating powder samples at a rate of 10 °C min^–1^ under flowing oxygen, using a PerkinElmer TGA 8000, with the exhaust gases monitored using a Hiden Analytical HPR-20 EGA mass spectrometer. AC and DC magnetization data were collected using a Quantum Design MPMS-3 SQUID magnetometer from samples contained in gelatin capsules.

X-ray absorption spectroscopy (XAS) data were collected using instrument B18 at the Diamond Light Source. The measurements were carried out using the Pt-coated branch of the collimating and focusing mirrors, a Si(111) double-crystal monochromator, and a pair of harmonic rejection mirrors. The size of the beam at the sample position was approximately 600 μm × 700 μm. The data were collected from self-supported pellets at the Mn–K edge (6539 eV) in transmission mode with a step size equivalent to 0.25 eV. Analysis of the data was performed using the DEMETER software package.^11^ The data were normalized with a linear pre-edge and polynomial postedge background subtracted from the raw ln(*I*_t_/*I*_0_) data.

## Results

### Characterization of Sr_2_Mn_0.5_Ir_0.5_O_4_

SXRD data collected from Sr_2_Mn_0.5_Ir_0.5_O_4_ could be indexed using a body-centered, tetragonal unit cell (*a* = 3.861 Å, c = 12.536 Å) with reflection conditions consistent with the *I*4/*mmm* space group. A Mn/Ir cation-disordered structural model for Sr_2_Mn_0.5_Ir_0.5_O_4_ was constructed based on the previously reported *n* = 1 Ruddlesden–Popper phase, Sr_4_CoIrO_8_,^11^ and refined against the SXRD data to achieve a good fit, as described in the Supporting Information.

### Reactivity of Sr_2_Mn_0.5_Ir_0.5_O_4_ with CaH_2_ and LiH

Small scale test reactions between Sr_2_Mn_0.5_Ir_0.5_O_4_ and CaH_2_, performed in the temperature range 300 < *T*/°C < 450, as described above, revealed no reaction occurred below 350 °C, and nontopochemical decomposition reactions occurred at 370 °C. Therefore, a large sample for characterization by NPD data (Sample 1) was prepared by mixing 1.7 g of Sr_2_Mn_0.5_Ir_0.5_O_4_ with 1 mol equivalent of CaH_2_, sealing the reaction mixture in an evacuated Pyrex ampule and heating for 7 days at 360 °C, with a heating rate of 1 °C min^–1^. CaO was then removed from the sample, as described above.

In contrast, small test reactions between Sr_2_Mn_0.5_Ir_0.5_O_4_ and LiH, performed in the temperature range 300 < *T*/°C < 450, as described above, revealed no reaction occurred below 375 °C, and nontopochemical decomposition reaction occurred at 410 °C. Therefore, a large sample for characterization by NPD data (Sample 2) was prepared by mixing 1.7 g of Sr_2_Mn_0.5_Ir_0.5_O_4_ with 4.5 mol equivalents of LiH, sealing the reaction mixture in an evacuated Pyrex ampule and heating for 7 days at 400 °C, with a heating rate of 1 °C min^–1^. Li_2_O and LiH were then removed from the sample, as described above.

### Characterization of Sample 1 – Sr_2_Mn_0.5_Ir_0.5_O_3.25_H_0.75_

SXRD data collected from the CaH_2_-reduced sample (Sample 1) could be indexed using a body-centered tetragonal unit cell (*a* = 3.789 Å, *c* = 12.806 Å) with reflection conditions consistent with the *I*4/*mmm* space group, indicating a topochemical transformation of Sr_2_Mn_0.5_Ir_0.5_O_4_ had occurred.

Thermogravimetric data collected while heating Sample 1 under flowing oxygen indicate a mass gain of 2.2%, consistent with an initial composition of Sr_2_Mn_0.5_Ir_0.5_O_3.49_. However, analysis of the exhaust gas reveals the release of water during oxidation, indicating that Sample 1 is in fact an oxyhydride of composition Sr_2_Mn_0.5_Ir_0.5_O_4–*x*_H_*y*_, as detailed in the Supporting Information.

**Figure 1:**
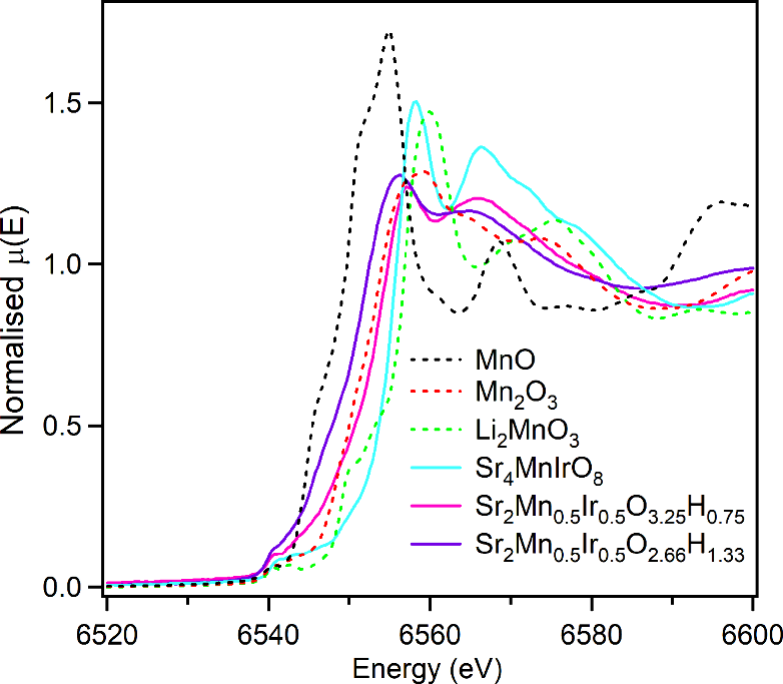
XANES data collected from the Mn K-edges of Sr_2_Mn_0.5_Ir_0.5_O_4_, Sr_2_Mn_0.5_Ir_0.5_O_3.25_H_0.75_ (Sample 1), Sr_2_Mn_0.5_Ir_0.5_O_2.66_H_1.33_ (Sample 2), MnO, Mn_2_O_3_, and Li_2_MnO_3_.

XANES data collected at the Mn–K edge from Sr_2_Mn_0.5_Ir_0.5_O_4_, Sample 1, Mn_2_O_3_ and Li_2_MnO_3_ (Mn^3+^ and Mn^4+^ standards respectively) are shown in Figure 1. These data show that the absorption edge from Sample 1 is at lower energy than that of Sr_2_Mn_0.5_Ir_0.5_O_4_, lying between the edges of the two standards, indicating a manganese oxidation state between Mn^3+^ and Mn^4+^.

**Figure 2:**
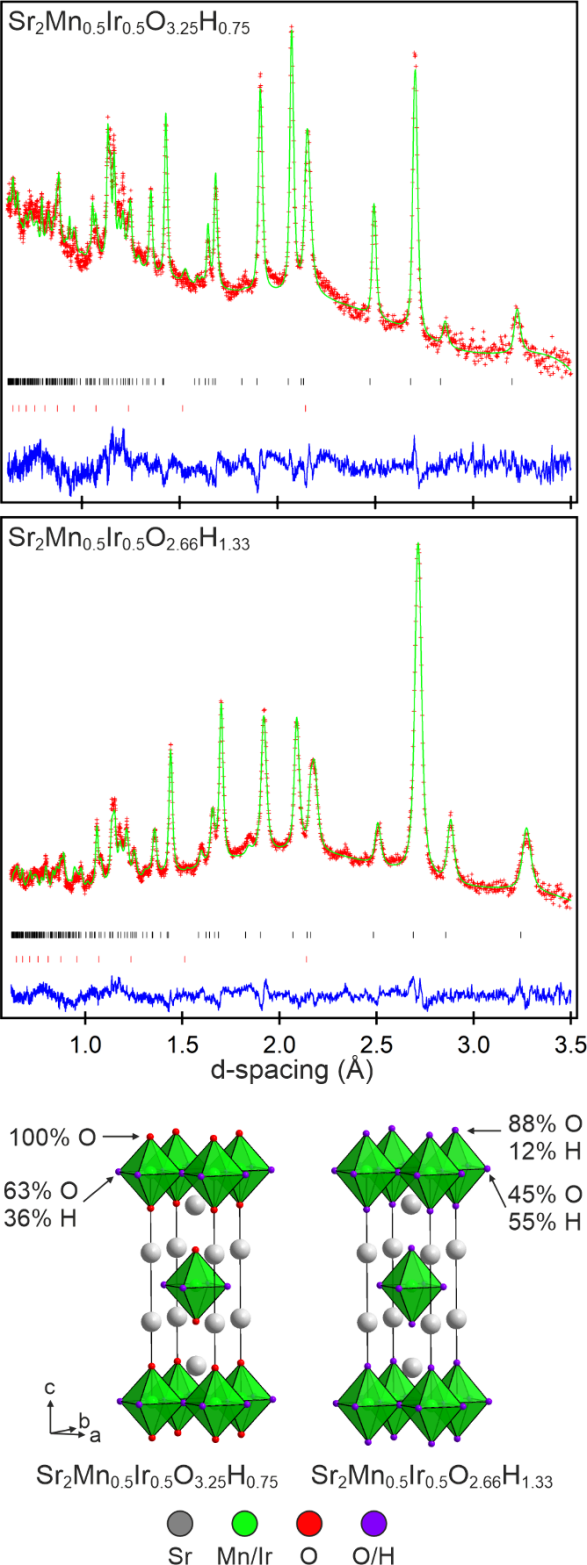
Observed calculated and difference plots from the structural refinement of Sr_2_Mn_0.5_Ir_0.5_O_3.25_H_0.75_ (top) and Sr_2_Mn_0.5_Ir_0.5_O_2.66_H_1.33_ (middle) against NPD data. Black tick marks indicate peak positions of the main phase, and red ticks indicate contributions from the vanadium sample holder. (bottom) Structures of Sr_2_Mn_0.5_Ir_0.5_O_3.25_H_0.75_ and Sr_2_Mn_0.5_Ir_0.5_O_2.66_H_1.33_.

NPD data collected from Sample 1 could also be indexed using a body-centered tetragonal unit cell. Thus, a model based on the structure of Sr_2_Mn_0.5_Ir_0.5_O_4_ was refined against these data with particular attention focused on the anion occupancies. Initially an “all-oxide” model was refined against the data, which converged readily, yielding a model with full occupancy of the O2 “axial” site and an occupancy of 0.393(8) for the O1 “equatorial” site, to give an overall composition of Sr_2_Mn_0.5_Ir_0.5_O_2.79_. This composition is unreasonable, in terms of both transition-metal oxidation state (requires a combined Mn + Ir oxidation state of 3.14) and coordination number (requires an average transition metal coordination number less than 4). Therefore, the model was changed to an oxyhydride system in which the O1 anion site was occupied by either oxide or hydride ions with a combined occupancy of 1. This model converged readily to give a 0.631(5):0.369(5) O:H occupancy of the O1 site, and an overall composition of Sr_2_Mn_0.5_Ir_0.5_O_3.26_H_0.74_. Full details of the structural refinement are given in Table 1, with plots of the fitted data shown in Figure 2 and Figure S3, and a representation of the refined structure of Sr_2_Mn_0.5_Ir_0.5_O_3.26_H_0.74_ shown in Figure 2.

**Table 1:** Parameters from the Structural Refinement of Sr_2_Mn_0.5_Ir_0.5_O_3.25_H_0.75_ (Sample 1) against NPD Data Collected at Room Temperature

Atom	*x*	*y*	*z*	Fraction	Biso (Å^2^)
Sr	0	0	0.3497(4)	1	1.0(1)
Mn/Ir	0	0	0	0.5/0.5	8.7(5)
O(1)/H(1)	0	1/2	0	0.631(5)/0.369(5)	0.2(1)
O(2)	0	0	0.1555(5)	1	0.9(1)
Sr_2_Mn_0.5_Ir_0.5_O_3.26(1)_H_0.73(1)_, space group *I*4/*mmm* (# 139)					
*a* = 3.7913(4) Å, *c* = 12.795(2) Å, volume = 183.92(5) Å^3^					
Formula weight = 351.57 g mol^–1^, *Z* = 2					
Radiation source: Time of Flight Neutron					
Temperature: 298 K					
*R*_*wp*_ = 2.39%, *R*_*p*_ = 1.87%, *R*_Bragg_ = 1.35%					

The refined composition of Sample 1, which we will henceforth refer to as Sr_2_Mn_0.5_Ir_0.5_O_3.25_H_0.75_, has a lower oxygen content than that indicated by the TGA data. However, given the neutron scattering lengths of oxide (5.80 fm) and hydride (−3.73 fm),^12^ this is the highest oxygen content model consistent with the NPD data, suggesting the TGA data overestimate the oxygen content, presumably due to incomplete reoxidation.

### Characterization of Sample 2 – Sr_2_Mn_0.5_Ir_0.5_O_2.66_H_1.33_

SXRD data collected from the LiH-reduced sample (Sample 2) could be indexed using a body-centered tetragonal unit cell (*a* = 3.807 Å, *c* = 12.977 Å) with reflection conditions consistent with the *I*4/*mmm* space group, indicating that a topochemical transformation of Sr_2_Mn_0.5_Ir_0.5_O_4_ had occurred.

Thermogravimetric data collected while heating Sample 2 under flowing oxygen indicate a mass gain of 5.5%, accompanied by the release of water, consistent with an initial composition of Sr_2_Mn_0.5_Ir_0.5_O_2.75_H_*y*_ as detailed in the Supporting Information. XANES data collected at the Mn–K edge from Sr_2_Mn_0.5_Ir_0.5_O_4_, Sample 2, MnO, and Mn_2_O_3_ (Mn^2+^ and Mn^3+^ standards respectively) are shown in Figure 1. These data show that the absorption edge from Sample 2 is at lower energy than that of Sr_2_Mn_0.5_Ir_0.5_O_4_, lying between the edges of the two standards, indicating a manganese oxidation state between Mn^2+^ and Mn^3+^.

NPD data collected from Sample 2 were refined by using the same strategy described for Sample 1. Initially an “all-oxide” model based on the structure of Sr_2_Mn_0.5_Ir_0.5_O_4_ was refined against the NPD data to yield a model in which the O1 and O2 anion sites had occupancies of 0.799(6) and 0.101(3), respectively, and an overall composition of Sr_2_Mn_0.5_Ir_0.5_O_1.8_. This model was then converted to an oxyhydride system in which the O1 and the O2 anion sites were occupied by a mixture of oxide and hydride ions, with combined occupancies of 1. This model converged readily to give a 0.456(2): 0.544(2) O:H occupancy for the O1 equatorial site and a 0.878(3):0.122(3) O:H occupancy for the O2 axial site, with an overall composition of Sr_2_Mn_0.5_Ir_0.5_O_2.66_H_1.33_. Full details of the structural refinement are given in Table 2, with plots of the fitted data shown in Figure 2 and Figure S5 and a representation of the refined structure of Sr_2_Mn_0.5_Ir_0.5_O_2.66_H_1.33_ shown in Figure 2. Again, the oxygen content of the model refined against the NPD data is less than indicated by the TGA data, which we again attribute to incomplete oxidation during the TGA measurement.

**Table 2:** Parameters from the Structural Refinement of Sr_2_Mn_0.5_Ir_0.5_O_2.66_H_1.33_ (Sample 2) against NPD Data Collected at Room Temperature

Atom	*x*	*y*	*z*	Fraction	Biso (Å^2^)
Sr	0	0	0.3519(1)	1	0.96(6)
Mn/Ir	0	0	0	0.5/0.5	4.4(2)
O(1)/H(1)	0	1/2	0	0.456(2)/0.544(2)	0.18(5)
O(2)/H(2)	0	0	0.1636(2)	0.878(3)/0.122(3)	0.18(5)
Sr_2_Mn_0.5_Ir_0.5_O_2.66(1)_H_1.33(1)_, space group *I*4/*mmm* (# 139)					
*a* = 3.8031(4) Å, *c* = 12.969(1) Å, volume = 187.58(4) Å^3^					
Formula weight = 342.88 g mol^–1^, *Z* = 2					
Radiation source: Time of Flight Neutron					
Temperature: 298 K					
*R*_*wp*_ = 1.37%, *R*_*p*_ = 1.07%, *R*_Bragg_ = 1.45%					

### Magnetic Characterization

**Figure 3:**
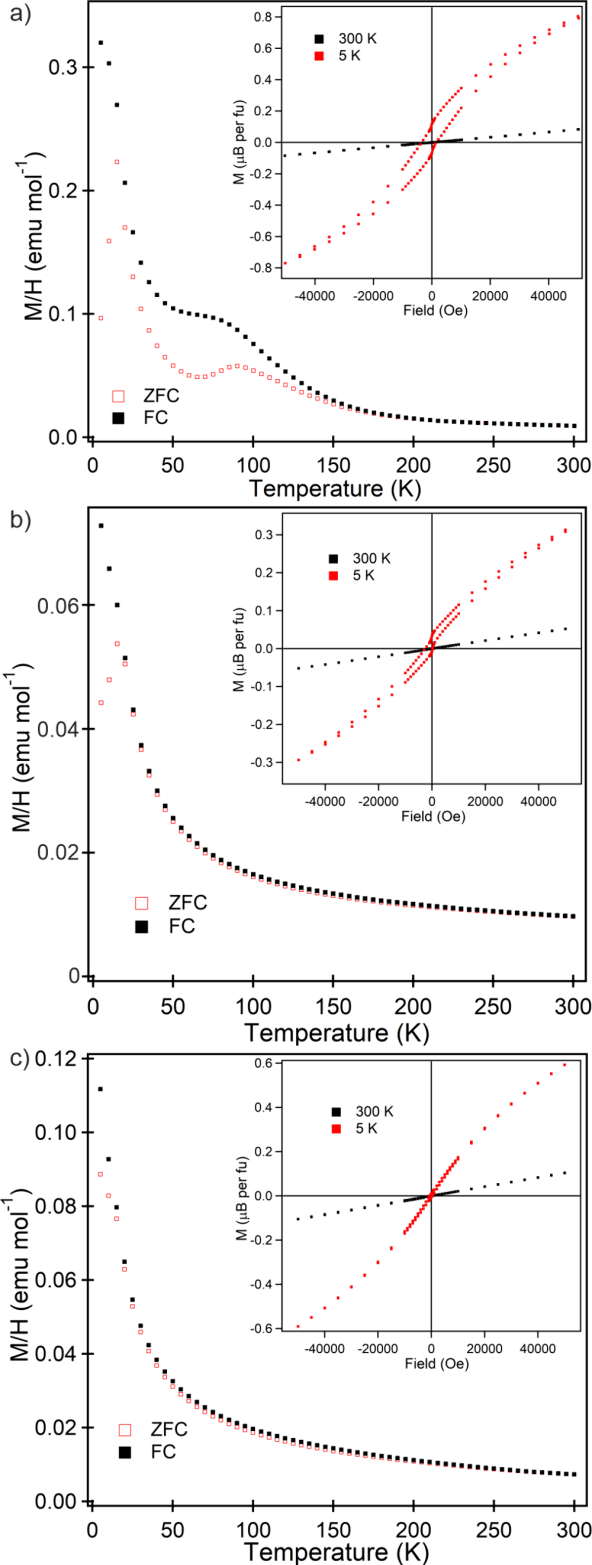
ZFC and FC magnetization data collected in an applied field of 100 Oe from a) Sr_2_Mn_0.5_Ir_0.5_O_4_, b) Sr_2_Mn_0.5_Ir_0.5_O_3.25_H_0.75_, and c) Sr_2_Mn_0.5_Ir_0.5_O_2.66_H_1.33_. Insets show magnetization-field data for the respective samples.

**Figure 4:**
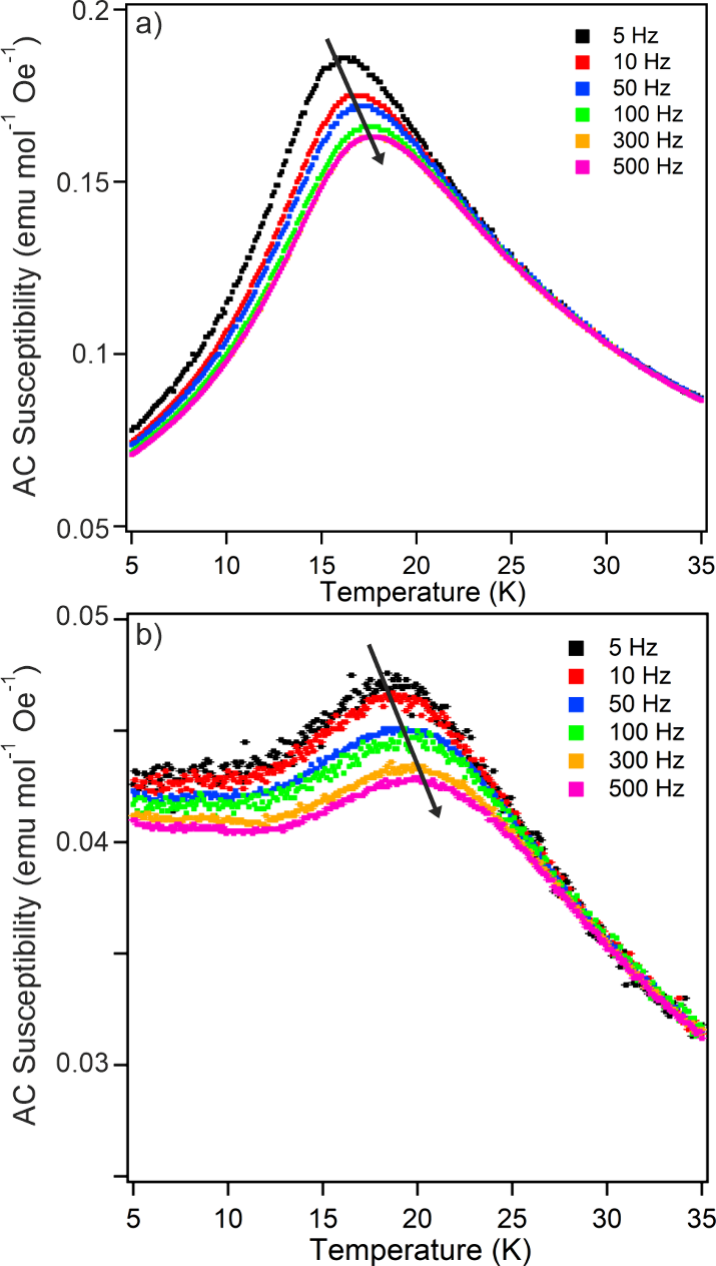
AC susceptibility collected as a function of temperature from a) Sr_2_Mn_0.5_Ir_0.5_O_4_ and b) Sr_2_Mn_0.5_Ir_0.5_O_3.25_H_0.75_.

Zero-field cooled (ZFC) and field-cooled (FC), DC magnetization data collected from Sr_2_Mn_0.5_Ir_0.5_O_4_ in an applied field of 100 Oe (Figure 3a) cannot be fit to the Curie–Weiss law over any temperature range. On cooling, the ZFC and FC data diverge below 200 K, with the ZFC data exhibiting local maxima at 90 and 15 K. Magnetization-field data collected from Sr_2_Mn_0.5_Ir_0.5_O_4_ at 300 K are linear and pass through the origin (Figure 3a). In contrast, magnetization-field data collected at 5 K, after cooling in an applied field of 5 T, exhibit hysteresis and are displaced from the origin, suggesting spin-glass behavior. AC susceptibility data (Figure 4a) show a strong frequency dependence around the 15 K feature, indicating that this is a glass-freezing transition.

DC magnetization data collected from Sr_2_Mn_0.5_Ir_0.5_O_3.25_H_0.75_ (Sample 1) can be fit to the Curie–Weiss law in the range 40 < *T*/K < 300 as shown in Figure 3b. However, the Curie constant extracted (98 cm^3^ K mol^–1^) is unphysically large, suggesting strong cooperative behavior of the local Mn and Ir spins, rather than a “simple” paramagnetic state. The ZFC and FC data diverge below *T* = 15 K, and again AC susceptibility data (Figure 4b) show a strong frequency dependence around this temperature, indicating this is a glass-freezing transition.

DC magnetization data collected from Sr_2_Mn_0.5_Ir_0.5_O_2.66_H_1.33_ (Sample 2) can be fit to the Curie–Weiss law in the range 25 < *T*/K < 300 as shown in Figure 3c. Again, the Curie constant extracted (244 cm^3^ K mol^–1^) is too unphysically large to be from a “simple” paramagnetic state. The ZFC and FC data diverge weakly below *T* ∼ 70 K but do not show any obvious anomalies indicative of magnetic transitions.

## Discussion

### Phase Stability

Sr_2_Mn_0.5_Ir_0.5_O_4_ adopts a B-site disordered, *n* = 1 Ruddlesden–Popper structure in which both the manganese and iridium centers adopt the +4 oxidation state. This phase can therefore be thought of as an analogue of the B-site disordered perovskite phase, CaMn_0.5_Ir_0.5_O_3_.^13^ Reaction of Sr_2_Mn_0.5_Ir_0.5_O_4_ with LiH or CaH_2_ in a sealed system occurs via topochemical hydride-for-oxide anion-exchange, to yield Sr_2_Mn_0.5_Ir_0.5_O_4–*x*_H_*x*_ oxyhydride phases–to our knowledge the first reported examples of iridium-containing oxyhydride compounds.

The conversion of Sr_2_Mn_0.5_Ir_0.5_O_4_ to Sr_2_Mn_0.5_Ir_0.5_O_4–*x*_H_*x*_ phases appears to proceed without the formation of anion-deficient, all-oxide, Sr_2_Mn_0.5_Ir_0.5_O_4–*x*_ intermediates. This behavior contrasts strongly with Mn-only oxides such as Sr_2_MnO_4_ and LaSrMnO_4_, which react with CaH_2_ via topochemical anion deintercalation, to form the anion-deficient oxides SrMnO_3.65_ and LaSrMnO_3.5_ respectively,^14,15^ with no evidence of oxyhydride formation or further reaction.

The differing behavior of Sr_2_Mn_0.5_Ir_0.5_O_4_ and Sr_2_MnO_4_ on reaction with CaH_2_ is attributed to the presence of iridium, which as a 5*d* transition-metal forms much stronger σ-type M-H bonds than the 3*d* transition-metal Mn. We propose that these strong Ir–H σ-bonds stabilize the Sr_2_Mn_0.5_Ir_0.5_O_4–*x*_H_*x*_ phases in a manner analogous to the “covalent stabilization” afforded to oxyhydride phases containing the 4*d* transition-metals Rh and Ru, in phases such as La_0.5_Sr_1.5_Mn_0.5_Rh_0.5_O_6_H_2_ or LaSr_3_NiRuO_4_H_4_.^4,9^

Further support for the “covalent stabilization” of Sr_2_Mn_0.5_Ir_0.5_O_4–*x*_H_*x*_ phases by strong Ir–H σ-bonds comes from the observed oxidation state of the Ir centers in these compounds. Mn K-edge XANES data from Sr_2_Mn_0.5_Ir_0.5_O_3.25_H_0.75_ and Sr_2_Mn_0.5_Ir_0.5_O_2.66_H_1.33_ (Figure 1) are consistent with Mn^3+^Ir^3+/4+^ and Mn^2+/3+^Ir^3+^ oxidation state combinations, respectively. The presence of d^6^ Ir^3+^ centers is entirely consistent with the “covalent stabilization” of the oxyhydride phases, as the low-spin configuration adopted by these centers maximizes the M-H σ-bond strength in these materials, as also observed for Rh^3+^ and Ru^2+^ centers in analogous oxyhydride phases.^4,9^

### Anion Distribution

One area where Sr_2_Mn_0.5_Ir_0.5_O_4–*x*_H_*x*_ phases are strikingly different from previously reported transition-metal oxyhydrides based on the Ruddlesden–Popper structure is in the distribution of the hydride ions within the anion framework. As noted above, reaction between Sr_2_Mn_0.5_Ir_0.5_O_4_ and CaH_2_ yields Sr_2_Mn_0.5_Ir_0.5_O_3.25_H_0.75_ in which three-eighths of the “equatorial” oxide ions have been replaced by hydride ions, as shown in Figure 2. Reaction between Sr_2_Mn_0.5_Ir_0.5_O_4_ and LiH goes further to yield Sr_2_Mn_0.5_Ir_0.5_O_2.66_H_1.33_ in which approximately half the equatorial oxide ions and ∼12% of the “axial” oxide ions are replaced by hydride ions. This replacement of axial oxide ions with hydride ions is highly unusual as the vast majority of transition-metal oxyhydrides with Ruddlesden–Popper structures only have hydride ions within equatorial anion sites.^16^

The observed preference for locating hydride ions within the equatorial sites of A_2_BO_*x*_H_*y*_ Ruddlesden–Popper oxyhydride phases mirrors the observed preference to also locate anion-vacancies within the equatorial anion sites of A_2_BO_4–*x*_ anion deficient oxides.^17,18^ Both of these structural preferences can be rationalized by noting that the equatorial anions in A_2_BX_4_ phases reside within an A_4_B_2_ coordination environment, compared to the A_5_B environment of the axial anions. Removing the O^2–^ ions from the equatorial site, or replacing them with H^–^ ions, lowers the bond valence sum of the B-cations more (changes more B-X interactions) than making the same change to the axial site. As a consequence, making changes at the equatorial sites leads to a modified phase with lower lattice strain, as explained in detail for the anion-deficient case previously.^17^

**Figure 5:**
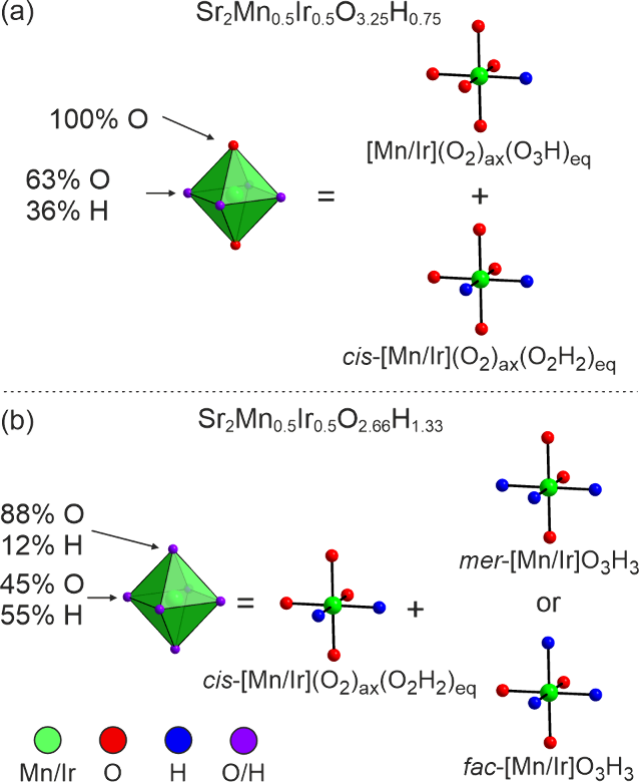
Local metal coordinations in (a) Sr_2_Mn_0.5_Ir_0.5_O_3.25_H_0.75_ and (b) Sr_2_Mn_0.5_Ir_0.5_O_2.66_H_1.33_.

We attribute the presence of hydride ions on the axial sites of Sr_2_Mn_0.5_Ir_0.5_O_2.66_H_1.33_, and the associated “violation” of the normal “equatorial hydride” structural selectivity preference, to a combination of factors. First, hydride ions in transition-metal oxyhydrides exhibit a strong *trans*-influence, which in the absence of stabilizing d-electron counts on the transition-metal centers (d^2^, high spin d^7^) disfavors anion configurations in which the strongly σ-donating hydride ions are *trans* to each other.^19^ In the case of Sr_2_Mn_0.5_Ir_0.5_O_3.25_H_0.75_ this drive to avoid *trans* arrangements of hydride ions around the Mn and Ir centers is compatible with the equatorial-hydride structural preference, as the disordered structure of Sr_2_Mn_0.5_Ir_0.5_O_3.25_H_0.75_ described in Table 1 can be deconvoluted into a 1:1 combination of [Mn/Ir](O_2_)_axial_(O_3_H)_equatorial_ and *cis*-[Mn/Ir](O_2_)_axial_(O_2_H_2_)_equatorial_ local configurations, as shown in Figure 5a. However, as the level of hydride-for-oxide substitution increases to yield phases of composition Sr_2_Mn_0.5_Ir_0.5_O_4–*x*_H_*x*_ with *x* > 2, these two structural preferences come into conflict, as these hydride rich compositions require the presence of [Mn/Ir]O_3_H_3_ local units, which must either adopt a *mer* configuration (Figure 5b), which respects the preference for locating hydride ions in equatorial sites, but contradicts the strong *trans-*influence of the hydride ions, or the [Mn/Ir]O_3_H_3_ units adopt a *fac* configuration (Figure 5b) which respects the strong *trans-*influence, but not the preference for locating hydride ions in equatorial sites. Thus, it can be seen that the *trans*-influence of the hydride ions weakens the preference to locate oxide ions in equatorial anion sites in phases with a high hydride concentration, and this effect is enhanced by the strong M-H σ-bonding of the 5d metal iridium.

A second factor which acts to overcome the equatorial-hydride structural preference can be seen by noting that *fac*-MO_3_H_3_ centers have approximate *C*_3*v*_ point symmetry which helps to retain the pseudodegeneracy of the d_*xz*_, d_*yz*_, and d_*xy*_ orbitals of the central metal. In the case of *fac*-IrO_3_H_3_ units this retention of orbital degeneracy enhances stabilization from spin–orbit coupling,^20,21^ and thus provides another interaction which opposes the equatorial-hydride structural preference.

We believe that a combination of these two factors: strong Ir–H *trans-*influence and spin–orbit stabilization of the *fac*-IrO_3_H_3_ units, weakens the equatorial-hydride structural preference, leading to the low concentration of hydride ions observed on the axial anion sites of Sr_2_Mn_0.5_Ir_0.5_O_2.66_H_1.33_. As described above, the presence of iridium strengthens the *trans*-influence of the M-H bonds, compared to analogous 3d or 4d transition-metal systems. In addition, the stabilization of *fac*-MO_3_H_3_ units via spin–orbit coupling is much stronger for 5d transition metals, compared to 3d or 4d systems, suggesting that it is the presence of 5d iridium in Sr_2_Mn_0.5_Ir_0.5_O_2.66_H_1.33_ that is ultimately responsible for the weakening of the equatorial-hydride structural preference and the location of some hydride ions on the axial anion sites of the phase.

### Magnetism

Sr_2_Mn_0.5_Ir_0.5_O_4_ and Sr_2_Mn_0.5_Ir_0.5_O_3.25_H_0.75_ adopt spin glass states below *T* ∼ 16 and 20 K, respectively. This behavior can be attributed to the combination of Mn/Ir structural disorder and frustration between nearest-neighbor and next-nearest-neighbor Mn–Mn, Mn–Ir, and Ir–Ir couplings.

In contrast, magnetization data collected from Sr_2_Mn_0.5_Ir_0.5_O_2.66_H_1.33_ show no indication of a transition to a magnetically ordered state. This can be rationalized by noting that all the iridium centers in Sr_2_Mn_0.5_Ir_0.5_O_2.66_H_1.33_ are in the +3 oxidation state, and adopt diamagnetic, low-spin d^6^ electronic configurations. The dilution of the system with 50% diamagnetic centers weakens the magnetic couplings present, suppressing any magnetic phase transition at least below *T* = 5 K, the lowest temperature we measured.

## Conclusion

Reaction of Sr_2_Mn_0.5_Ir_0.5_O_4_ with CaH_2_ or LiH leads to the formation of the oxyhydride phases Sr_2_Mn_0.5_Ir_0.5_O_3.25_H_0.75_ or Sr_2_Mn_0.5_Ir_0.5_O_2.66_H_1.33_ via topochemical anion exchange. The presence of Ir^3+^ centers in these phases suggests strong Ir–H σ-bonds “covalently stabilize” these metastable phases in a manner analogous to that observed for ruthenium and rhodium containing oxyhydrides. However, the strong *trans*-influence associated with these Ir–H σ-bonds, combined with the spin–orbit stabilization of *fac*-IrO_3_H_3_ local coordination, modifies the normal preference for locating hydride ions on the equatorial anions sites of Ruddlesden–Popper frameworks, allowing the location of a small number of hydride ions onto the axial anions sites of Sr_2_Mn_0.5_Ir_0.5_O_2.66_H_1.33_.

## Supporting Information Available

Structural characterization of Sr_2_Mn_0.5_Ir_0.5_O_4_; thermogravimetric data collected during the oxidation of Sr_2_Mn_0.5_Ir_0.5_O_3.25_H_0.75_ and Sr_2_Mn_0.5_Ir_0.5_O_2.66_H_1.33_; plots of all data from structural refinement of Sr_2_Mn_0.5_Ir_0.5_O_3.25_H_0.75_ and Sr_2_Mn_0.5_Ir_0.5_O_2.66_H_1.33_ (PDF)
