# Supplementary material for: Covalent Stabilization of the Iridium-Containing Oxyhydrides Sr2Mn0.5Ir0.5O3.25H0.75 and Sr2Mn0.5Ir0.5O2.66H1.33
Source: Inorg Chem. 2024 Nov 5;63(46):22308–14. doi: 10.1021/acs.inorgchem.4c04057 (PMC11577310; doi:10.1021/acs.inorgchem.4c04057)
Supplement: Supplementary file 1 — ic4c04057_si_001.pdf [file ic4c04057_si_001.pdf]

Covalent stabilization of the iridium-containing oxyhydrides $Sr_2Mn_{0.5}Ir_{0.5}O_{3.25}H_{0.75}$ and $Sr_2Mn_{0.5}Ir_{0.5}O_{2.66}H_{1.33}$

James Murrell,† and Michael A. Hayward †*.

† Department of Chemistry, University of Oxford, Inorganic Chemistry Laboratory, South Parks Road, Oxford, OX1 3QR, UK.

Table of Contents

1. Structural Characterisation of $Sr_2Mn_{0.5}Ir_{0.5}O_4$.

Figure S1. Observed calculated and difference plots from the structural refinement of $Sr_2Mn_{0.5}Ir_{0.5}O_4$ against SXRD data.

Table S1. Parameters from the structural refinement of $Sr_2Mn_{0.5}Ir_{0.5}O_4$ against SXRD data.

2. Characterisation of $Sr_2Mn_{0.5}Ir_{0.5}O_{3.25}H_{0.75}$ (Sample 1).

Figure S2. Thermogravimetric data (top) and m/z = 18, m/z = 2 mass-spectrum signals (bottom) collected as a function of temperature during the reoxidation of Sample 1 back to $Sr_2Mn_{0.5}Ir_{0.5}O_4$ under flowing oxygen.

Figure S3. Observed calculated and difference plots from the structural refinement of $Sr_2Mn_{0.5}Ir_{0.5}O_{3.25}H_{0.75}$ against NPD data.

3. Characterisation of $Sr_2Mn_{0.5}Ir_{0.5}O_{2.66}H_{1.33}$ (Sample 2).

Figure S4. Thermogravimetric data (top) and m/z = 18, m/z = 2 mass-spectrum signals (bottom) collected as a function of temperature during the reoxidation of Sample 2 back to $Sr_2Mn_{0.5}Ir_{0.5}O_4$ under flowing oxygen.

Figure S5. Observed calculated and difference plots from the structural refinement of $Sr_2Mn_{0.5}Ir_{0.5}O_{2.66}H_{1.33}$ against NPD data.

1. Structural Characterisation of $Sr_2Mn_{0.5}Ir_{0.5}O_4$.

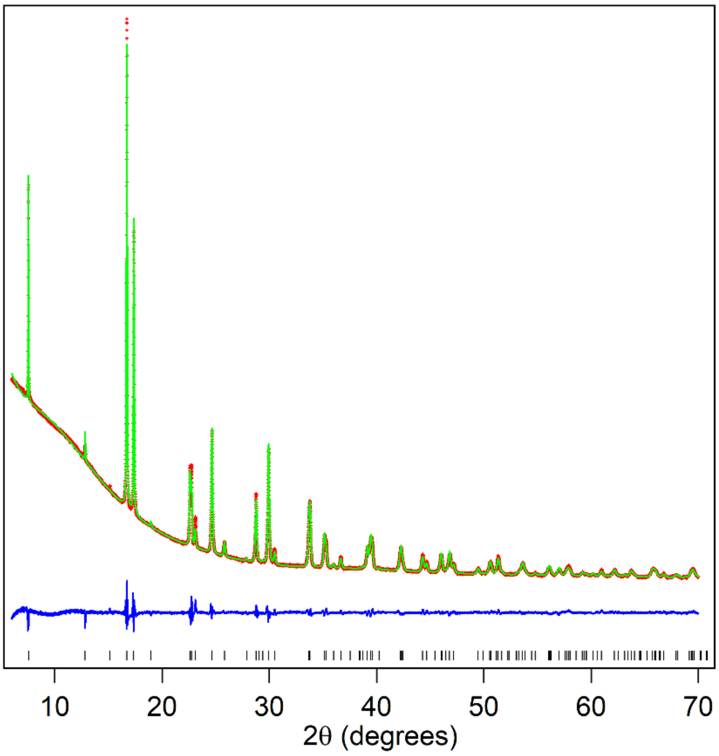

Figure S1. Observed calculated and difference plots from the structural refinement of $Sr_2Mn_{0.5}Ir_{0.5}O_4$ against SXRD data.

Atom	x	y	z	Fraction	Biso (Å^2)
Sr	0	0	0.35532(4)	1	0.86(1)
Mn/Ir	0	0	0	0.5/0.5	2.06(2)
O(1)	0	½	0	1	1.40(4)
O(2)	0	0	0.1604(3)	1	1.40(4)
$Sr_2Mn_{0.5}Ir_{0.5}O_4$, space group *I*4/*mmm* (# 139)<br>*a* = 3.86076(3) Å, *c* = 12.5364(2) Å, volume = 186.861(4) Å^3<br>Formula weight = 362.81 g mol^{-1}, Z = 2					
Radiation source: Synchrotron X-ray radiation (λ = 0.825 Å)<br>Temperature: 298 K<br>R_{wp} = 1.88%, R_p = 1.20%					

Table S1. Parameters from the structural refinement of $Sr_2Mn_{0.5}Ir_{0.5}O_4$ against SXRD data.

2. Characterisation of $Sr_2Mn_{0.5}Ir_{0.5}O_{3.25}H_{0.75}$ (Sample 1).

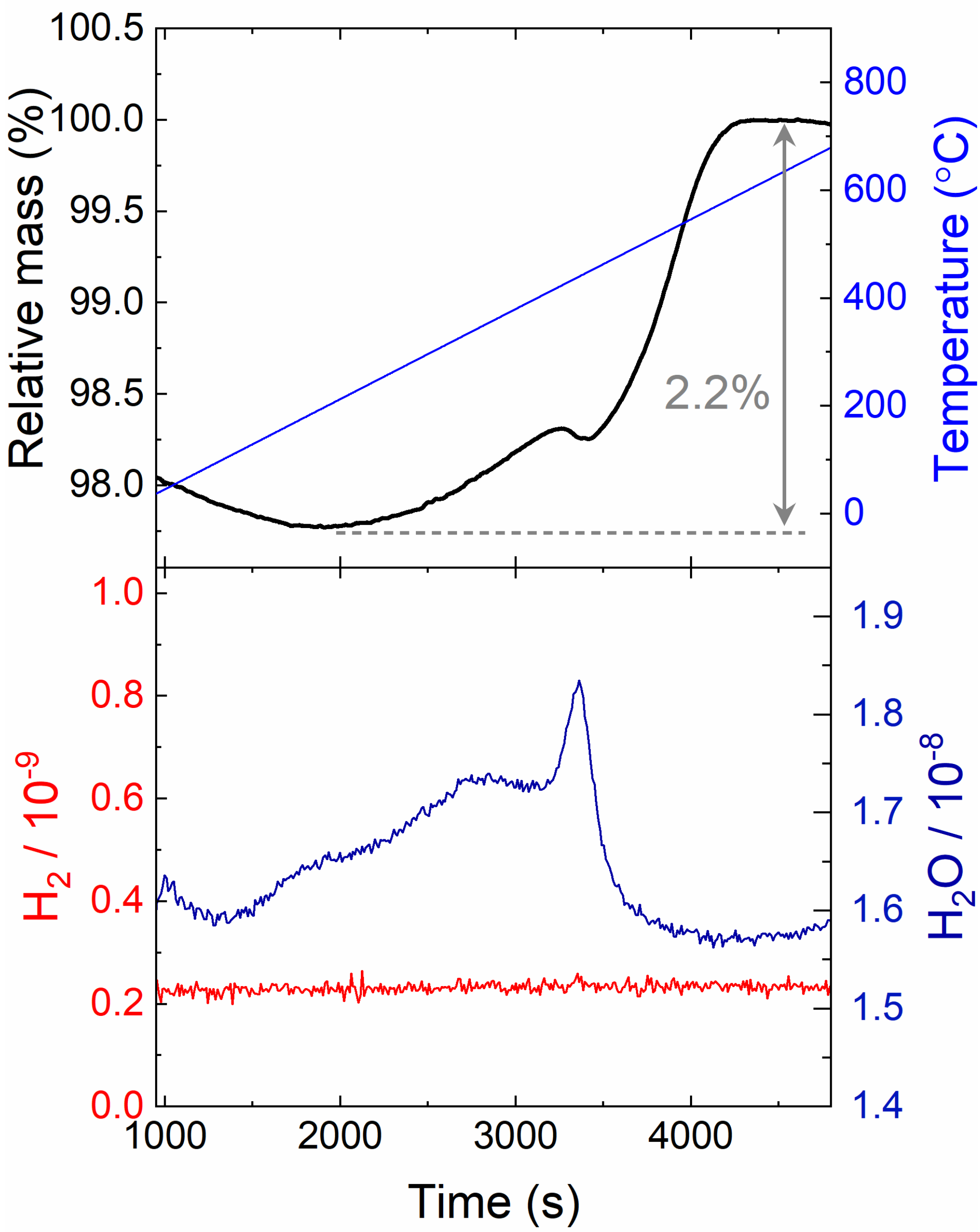

Figure S2. Thermogravimetric data (top) and m/z = 18, m/z = 2 mass-spectrum signals (bottom) collected as a function of temperature during the reoxidation of Sample 1 back to $Sr_2Mn_{0.5}Ir_{0.5}O_4$ under flowing oxygen.

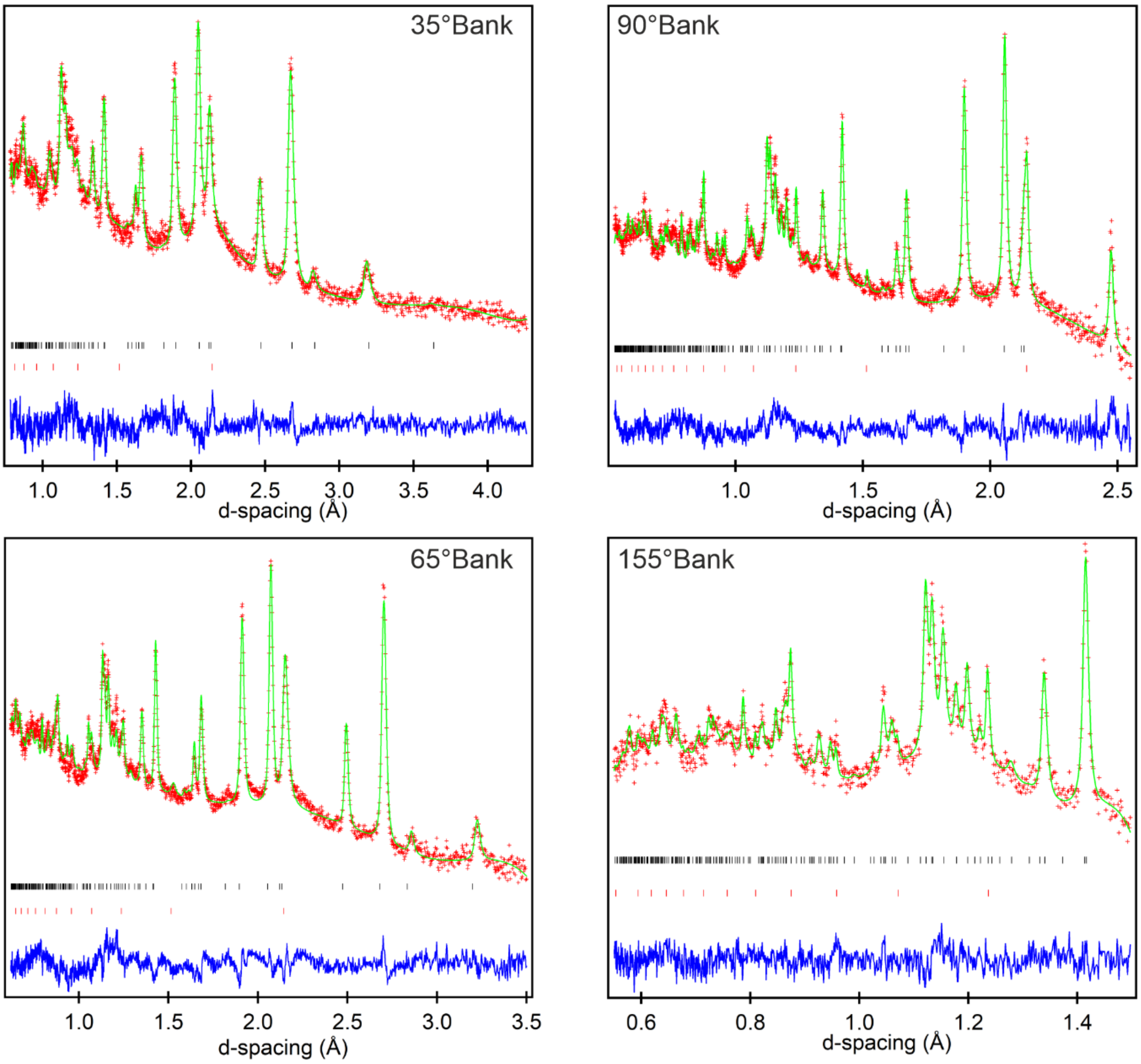

Figure S3. Observed calculated and difference plots from the structural refinement of $Sr_2Mn_{0.5}Ir_{0.5}O_{3.25}H_{0.75}$ against NPD data. Black tick marks indicate peak positions of the main phase, red ticks indicate contributions from the vanadium sample holder.

3. Characterisation of $Sr_2Mn_{0.5}Ir_{0.5}O_{2.66}H_{1.33}$ (Sample 2).

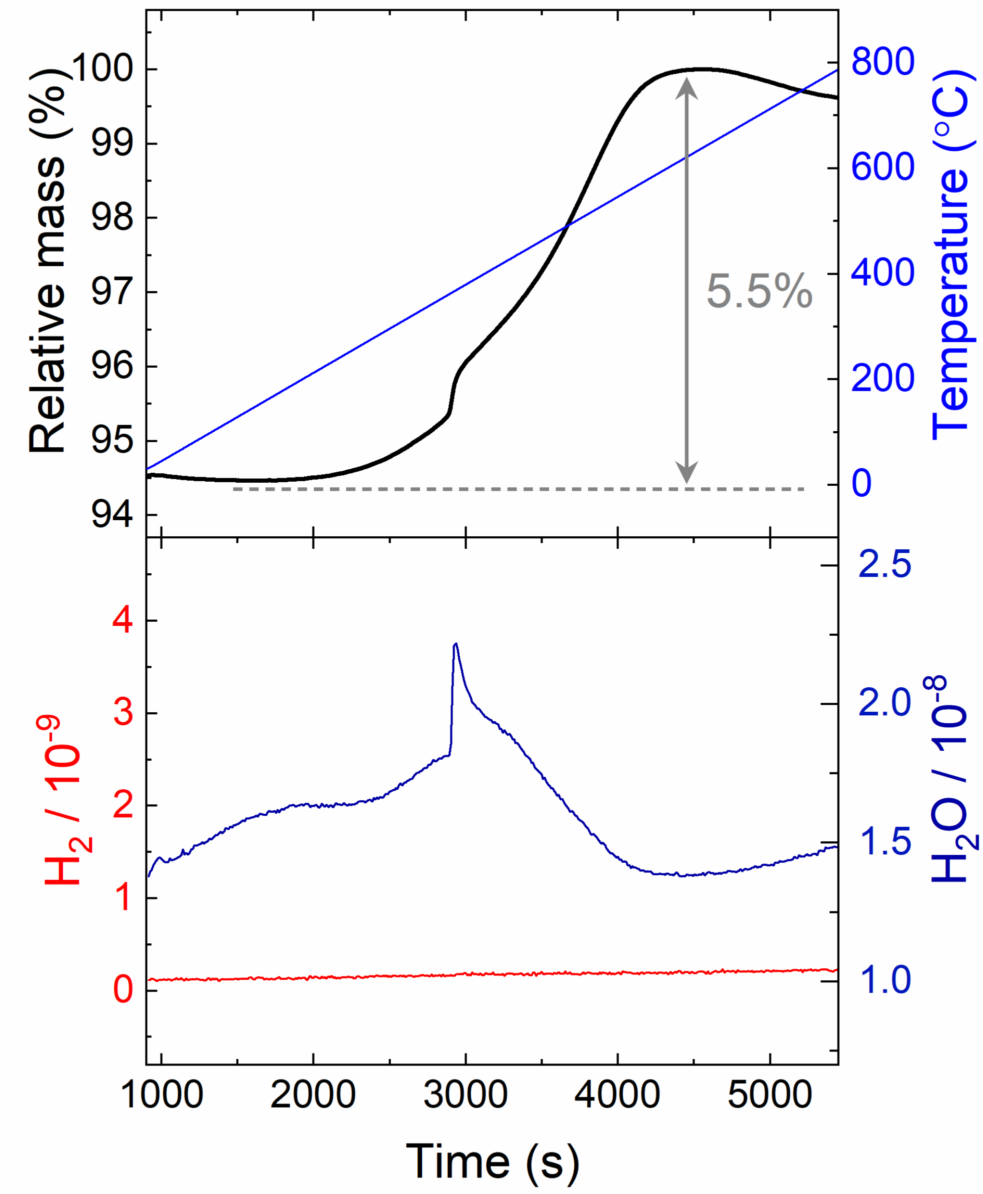

Figure S4. Thermogravimetric data (top) and m/z = 18, m/z = 2 mass-spectrum signals (bottom) collected as a function of temperature during the reoxidation of Sample 2 back to $Sr_2Mn_{0.5}Ir_{0.5}O_4$ under flowing oxygen.

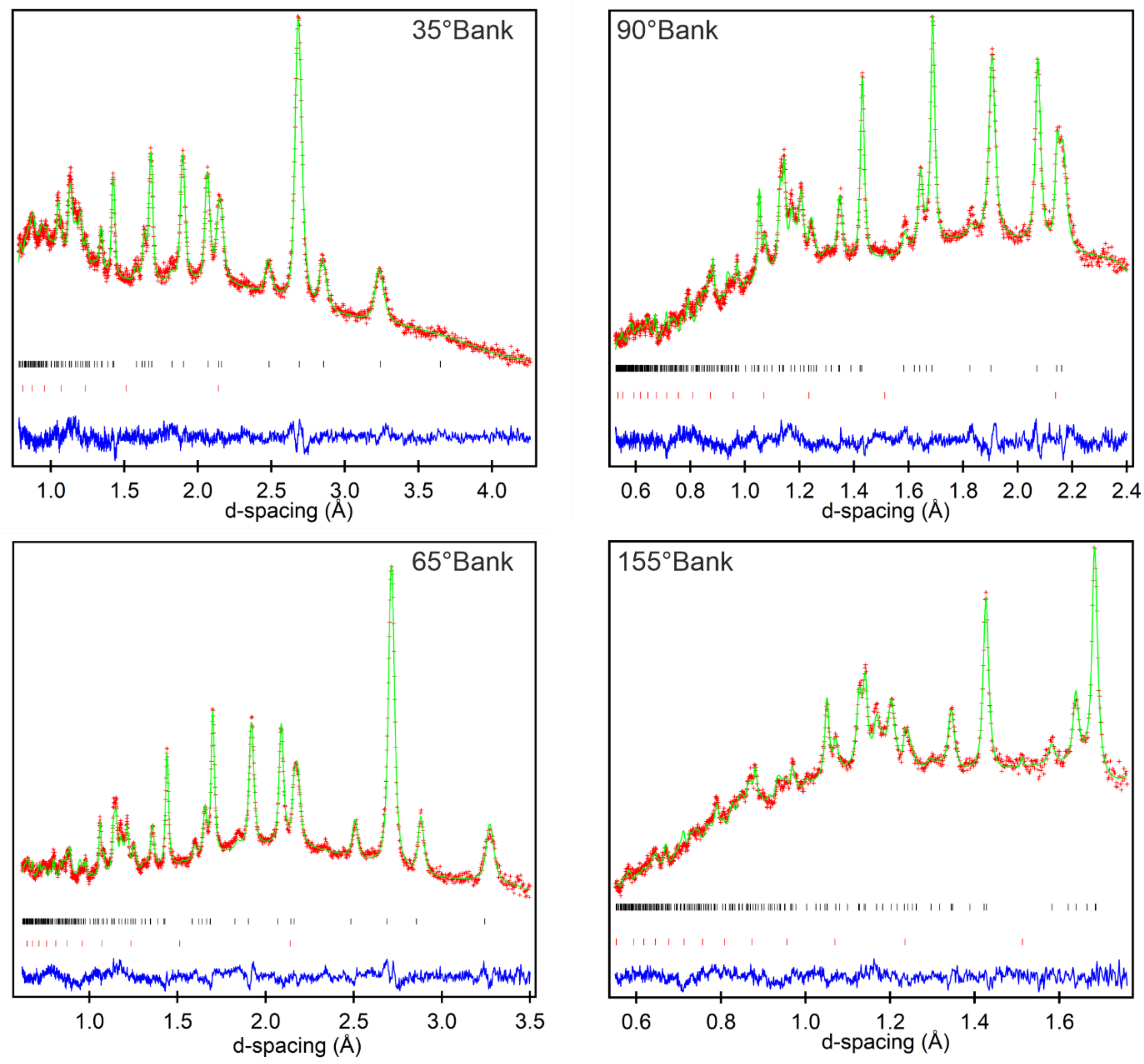

Figure S5. Observed calculated and difference plots from the structural refinement of $Sr_2Mn_{0.5}Ir_{0.5}O_{2.66}H_{1.33}$ against NPD data. Black tick marks indicate peak positions of the main phase, red ticks indicate contributions from the vanadium sample holder.
